# The Autonomization Principle in Vascularized Flaps: An Alternative Strategy for Composite Tissue Scaffold In Vivo Revascularization

**DOI:** 10.3390/bioengineering10121440

**Published:** 2023-12-18

**Authors:** Yanis Berkane, David M. Kostyra, Theodoros Chrelias, Mark A. Randolph, Alexandre G. Lellouch, Curtis L. Cetrulo, Korkut Uygun, Basak E. Uygun, Nicolas Bertheuil, Jérôme Duisit

**Affiliations:** 1 Department of Plastic, Reconstructive and Aesthetic Surgery, Rennes University Hospital Center, Rennes University, 16 Boulevard de Bulgarie, 35000 Rennes, Francetchrelias@gmail.com (T.C.); nbertheuil@gmail.com (N.B.); 2 Vascularized Composite Allotransplantation Laboratory, Massachusetts General Hospital, Harvard Medical School, 50 Blossom Street, Boston, MA 02114, USA; marandolph@mgh.harvard.edu (M.A.R.); alellouch@mgb.org (A.G.L.); ccetrulo@mgh.harvard.edu (C.L.C.J.); 3 Shriners Children’s Boston, 51 Blossom Street, Boston, MA 02114, USA; kuygun@mgh.harvard.edu (K.U.); basakuygun@mgh.harvard.edu (B.E.U.); 4 SITI Laboratory, UMR1236, INSERM, Rennes University, 2 Rue Henri le Guillou, 35000 Rennes, France; 5 Plastic Surgery Research Laboratory, Massachusetts General Hospital, Harvard Medical School, 50 Blossom Street, Boston, MA 02114, USA; dkostyra@mgh.harvard.edu; 6 Wellman Center for Photomedicine, Massachusetts General Hospital, Harvard Medical School, 50 Blossom Street, Boston, MA 02114, USA; 7 Center for Engineering in Medicine and Surgery, Massachusetts General Hospital, Harvard Medical School, 50 Blossom Street, Boston, MA 02114, USA; 8 IRIS Sud Hospitals, Rue Baron Lambert 38, 1040 Etterbeek, Belgium

**Keywords:** autonomization, autonomisation, flap neo-vascularization, flap revascularization, tissue engineering, flap bioengineering, tissue perfusion, decellularization, scaffold revascularization

## Abstract

Autonomization is a physiological process allowing a flap to develop *neo-vascularization* from the reconstructed wound bed. This phenomenon has been used since the early application of flap surgeries but still remains poorly understood. Reconstructive strategies have greatly evolved since, and fasciocutaneous flaps have progressively replaced muscle-based reconstructions, ensuring better functional outcomes with great reliability. However, plastic surgeons still encounter challenges in complex cases where conventional flap reconstruction reaches its limitations. Furthermore, emerging bioengineering applications, such as decellularized scaffolds allowing a complex extracellular matrix to be repopulated with autologous cells, also face the complexity of revascularization. The objective of this article is to gather evidence of autonomization phenomena. A systematic review of flap autonomization is then performed to document the minimum delay allowing this process. Finally, past and potential applications in bio- and tissue-engineering approaches are discussed, highlighting the potential for in vivo revascularization of acellular scaffolds.

## 1. Introduction

In modern reconstructive surgery, fasciocutaneous flaps have gradually become an alternative to classic muscle flaps due to a better outcome/morbidity balance [1,2,3]. In all cases, once transposed to the recipient site, flaps gradually develop capillary anastomoses with the wound bed until the established blood flow through this neo-vascularization is sufficient for its survival: this phenomenon is known as autonomization [4]. This principle has been used since antiquity, with the first descriptions of the forehead flap in India [5] and its still-relevant technique [6,7,8]. The process involved vascularized flap dissection, transposition to the recipient defect, and allowance of the autonomization process to occur in 3 to 6 weeks [7,8]. After this delay, the source vessels initially providing the flap blood supply can be divided, typically preceded by a clamp test. This last step assesses if the autonomization process is sufficient to ensure flap survival. Thereafter, the concept was extended to many reconstruction techniques: in face reconstruction, von Pfalzpaint and Tagliacozzi described the cross-arm flap for nose reconstruction [9,10,11], and Dufourmentel described chin reconstruction using a double-pedicled scalp flap [12], which is still used in modern plastic surgery. Later on, Burget and Menick described nasal reconstruction using a nasolabial flap, with a second step after 3 weeks [13]. In limb reconstruction, the McGregor technique has been described for the upper limbs [14] and the pedicled cross-leg flap for the lower limb [15]: these are prominent examples of using autonomization principles in fasciocutaneous flaps. They allow last-resort reconstructions with a certain robustness and outcome security and are still used today [16]. Although microsurgery and free flaps have mostly replaced these techniques in modern surgery, they can still be used for complex cases, proving that modern techniques continue to be inspired by and perfect these ancestral techniques instead of replacing them. For instance, some authors described the free cross-leg flap technique, combining the principles of transient pedicled flaps and microsurgery [17,18]. Others described using a wrist carrier for vascular support of a combined fibular and anterolateral flap to treat a vessel-depleted neck [19]. For each of the existing techniques, the delay in healing and neovascularization/autonomization has not been clearly studied and established, and still varies with authors. Most surgeons choose these durations according to the surgical site receiving reconstruction, based on the descriptions of the reference techniques over time. Thus, for head and neck reconstructions—these territories being highly vascularized—the transient pedicled flaps are usually detached after 3 weeks [6,20,21]. For limb reconstructions, the commonly accepted duration for autonomization is often more important, varying from 4 to 6 weeks [14,15,17,22]. Still, the mechanisms involved in the autonomization process seem poorly understood, mainly because of the low expected impact they would have in current clinical practice. Furthermore, despite growing knowledge and improved techniques for flap reconstruction, failure still occurs, and extremely complex cases still face a lack of optimal reconstructive solutions [23]. 

The growing field of regenerative medicine and tissue engineering could present novel applications of so-called old techniques like autonomization. At a time when research studies are exploring new applications of bioengineering in reconstructive surgery [24,25,26,27,28], the autonomization principle appears to be a cornerstone. Some authors looked into the revascularization of simple dermal matrices such as Alloderm or DermaCell, showing that this phenomenon could occur after 2 weeks in gingival augmentation [29] and likely sooner in skull base reconstruction [30]. Fast revascularization of these acellular materials is critical for preventing infection and for the overall objective of replacing autologous tissues. Capito et al. [31] showed early cellular infiltration and evident angiogenesis by day 7 in a subcutaneous use of diverse acellular matrices. Similarly, Menon et al. [32] showed that Alloderm does become vascularized when used for abdominal wall reconstruction. However, acellular dermal matrices are thin layers of extracellular components. They cannot be used for complex defect reconstruction, as a substitute for flaps or vascularized composite allotransplantation. Complex tissue scaffolds such as total face [24], ears [25], hands [33], and vascularized flaps [28] have already been described. The objective is to eventually use these scaffolds as a recipient matrix for autologous revascularization and subsequent recellularization [34,35]. Recellularization can be performed in vitro using various seeding techniques [36]. It can also be carried out partially in vivo. In this case, the main challenge will remain, as for native fasciocutaneous flaps, in ensuring adequate vascularization of the tissues to allow the survival of the different cell types reseeded within the scaffold. Several attempts to perform engineered re-endothelialization of the vascular tree have shown only poor results so far. An alternative could be using purely in vivo vascular autonomization from the wound bed and the wound margins to perform revascularization of complex engineered scaffolds. This is a unique feature of composite flaps, contrasting with engineered solid organs.

In order to achieve in vivo scaffold revascularization, more precise insights regarding its mechanisms and timeline are needed. Overall, to date, there is no consensus on the time frame for autonomization or on the factors promoting or delaying the autonomization process. The purpose of this work is, therefore, to perform a systematic review to report on autonomization physiology, with a focus on early autonomization of autologous fasciocutaneous flaps. This will enable a discussion of how it could be efficiently used for scaffold revascularization, eventually allowing applications in bioengineering approaches to complex reconstructions. 

## 2. Materials and Methods

We undertook this review in June 2023. First, we screened publications treating autonomization physiology and fasciocutaneous flap autonomization in animal models. Second, we performed a literature review to understand the sub-cited, commonly admitted autonomization delays in flaps with delayed pedicle division. Finally, we undertook a systematic review focusing on the early autonomization of fasciocutaneous flaps in clinics with no further intervention. This last step was performed following the Preferred Reporting Items for Systematic Reviews and Meta-Analysis (PRISMA) statement, updated in 2020 [37,38]. Our proceeding is AMSTAR-compliant (Assessing the Methodological Quality of Systematic Reviews) and is available online (PROSPERO Registration number CRD42022363596).

**Part 1: Autonomization physiology and animal studies.**

We used keywords such as “Flap”, “Autonomization”, “Neovascularization”, and their variations on the PubMed database, and screened all publications focusing on this phenomenon. We screened only articles using species other than humans. We included articles focused on fasciocutaneous flap integration and vascular autonomy, and thus related to autonomization. Because of the varied designations for this principle, most of the relevant articles were found while screening “similar articles”. 

**Part 2: Early evidence in humans and current standards for fasciocutaneous flap autonomization.**

We performed a literature review on Pubmed using keywords and Boolean operators as follows: “Flap”, “Autonomization”, “Neo-vascularization”, “Neovascularisation”, “Angiogenesis”. No time frame was selected (All time). We selected articles with a title showing a focus on the autonomization/neovascularization phenomenon. Several articles were found by screening “similar articles” and citations from publications of interest. The objective of this search was to summarize the current applications and commonly admitted autonomization delays in clinical practice. 

**Part 3: Systematic review of early flap autonomization in reconstructive surgery.**

We included published reports (original articles, randomized controlled trials, controlled clinical trials, retrospective or prospective observational studies, case reports, letters to the editor, comments, and technical descriptions) that provided data about early fasciocutaneous flap autonomization, free fasciocutaneous flap survival despite early failure of anastomoses, and fasciocutaneous flap survival after deliberate early division of the blood source in humans. The objective of this search was to report evidence of early autonomization when compared with the current timetable used in clinics, in order to leverage this principle to the optimum [6,16,39,40,41].

(a) **Search strategy**

**Figure 1:**
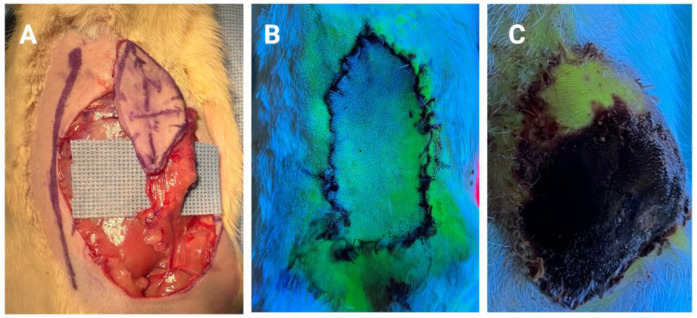
Early autonomization of the distal part of a rat superficial inferior epigastric artery (SIEA) fasciocutaneous flap. (**A**) SIEA flap harvested from distal to proximal, completely depending on the blood flow through the SIEA. (**B**) Immediate postoperative angiography (IV injection of fluorescein and Wood’s lamp) displaying poor vascularization of the distal tip of the flap. (**C**) Final aspect on postoperative day 10, after ligation of the SIEA vessels on POD5, showing subtotal necrosis of the lap. The distal part of the flap survived and showed optimal perfusion with subsequent angiography, proving autonomization from the divided SEIA vessels [44].

Two independent authors (Y.B and D.M.K.) performed the article screening process as follows. Final results were reached after discussion and a final consensus was found by the senior author (J.D.). Eligible studies were identified from the PubMed and Cochrane Library databases using the following keywords combined with Boolean operators: Title/Abstract: Flap **AND** (Neo-vascularization OR Neo-vascularisation OR Neovascularization OR Neovascularisation OR Autonomization OR Autonomisation OR Survival OR Salvage). Reference lists of selected articles were also manually examined to identify additional potentially eligible articles. The search strategy is summarized in Figure 1.

(b) **Exclusion criteria**
  (i) Excluded during title/abstract screening: studies lacking original data; studies with non-human subjects; studies in any language other than English or French; unavailable full manuscripts.
  (ii) Excluded during full-text analysis: articles describing flap failure without describing the vascular compromise; articles describing flap survival after surgical revision of the anastomosis; external intervention prior to pedicle division, or flap survival following a delayed vascular compromise later than 2 weeks for head, neck and hand] and 3 weeks for all other sites.
(c) **Data extraction**

Extracted data included: study design and characteristics; flap performed; type of fasciocutaneous flap; dimensions of the flap; recipient site; postoperative day of flow interruption; type of flow interruption (artery, vein, pedicle); patient history and characteristics: age, smoking status, diabetes, initial pathology, previous irradiation on the recipient site, infection of the recipient site; and flap partial loss. 

Data extraction was performed by two authors (Y.B. and D.M.K.), and the senior author (J.D.) helped to decide in case of a discrepancy.

(d) **Statistical analysis**

Quantitative variables were collected in Excel (v.16.36, Microsoft, Redmond, Washington) and transposed in Prism (v10.0.2, GraphPad Software, La Jolla, CA, USA). Descriptive statistics (mean, standard deviation) and multiple Student’s *t*-tests with Welch correction and Benjamini, Krieger, and Yekutieli’s two-stage linear step-up procedure were performed. Binary variables were analyzed in a contingency table, and Fisher’s exact test was performed. It was our intention to conduct a meta-analysis, but the lack of homogeneity in the study designs, the data selected by each author, and the many missing numerical values, led us to focus on sub-group analyses with multiple *t*-tests.

(e) **Bias assessment**

The authors followed the Joanna Briggs Institute (JBI) Critical Appraisal Checklist for Systematic Reviews [42]. The risk of bias was also assessed using the ROBIS tool (University of Bristol) [43]. Table A1 in Appendix A shows the overall risk of bias evaluated as low. 

## 3. Results

**Part 1:**

In 1985, Semashko et al. [45] focused on the autonomization process in superficial inferior epigastric artery (SIEA) fasciocutaneous flaps in rats. They used fluorescein angiography to quantify the flap vascularization at different locations over time. They showed that the distal tip of the flap was better vascularized than the proximal part, as soon as after 24 h, and that the whole flap could fully survive on its neo-vascularization with the recipient site after 5 days. We found similar results using a modified model [44], with an early autonomization of the distal tip in the rodent model (Figure 1). Other authors used the same model and described the use of ischemic preconditioning of the flap to optimize this autonomization process, mostly in rodent models [44,46,47]. A few publications studied neo-vascularization in fasciocutaneous flaps and reported a delay of 7–10 days for SIEA flaps in rats [48]. Some authors even assessed vascular endothelial growth factor (VEGF) gene therapy, or the addition of VEGF on the flap/wound bed interface, to improve the neo-vascularization time [49]. Angelos et al. showed that VEGF pDNA improved flap survivability following the early ligation of ventral flaps in irradiated rats [50]. However, those results need to be considered with care since rodent models are not always relevant to clinical applications. Indeed, the size and thickness of rat skin flaps are much less than in humans, and rat skin is poorly similar to human skin, in contrast with porcine models that are accepted as valid [51]. 

Towards generating data that is more clinically relevant than that gathered from rodent studies, Tsur et al. [52] investigated neo-vascularization time in flaps raised in swine by ligating the vascular pedicle between days 1 and 7 postoperatively. They demonstrated that the flaps could survive ligation as soon as 4 days postoperatively, and also found that neo-vascularization occurred from both the wound edges and bed. The neo-vascularization capabilities of the pedicle itself were also investigated, and the use of an expander was shown to enable neo-vascularization and the subsequent raising of a flap after the skin’s connection to the pedicle was interrupted [53]. A study by Young further investigated neo-vascularization by raising flaps too large for the pedicle to adequately perfuse the whole of the flap, thus creating areas of relative hypoxia [54]. These areas demonstrated faster and more dense neo-vascularization than the well-perfused areas of the flap, suggesting that hypoxia is a significant driver of neo-vascularization. Park et al. further expanded upon the work of Young by transferring flaps larger and smaller than the area perfused by the pedicle, as shown by intravenous fluorescein injection, to a separate wound bed [55]. Upon division of the pedicles 2 weeks later, the larger flaps were shown to have a significantly greater viable area as compared to the smaller flaps, providing further evidence that hypoxia improves neo-vascularization and thus flap survival after pedicle loss. 

Similar work was also carried out in rabbits, which represent good intermediate models between rodents and swine. Klöppel et al. showed that neo-vascularization after implantation of a skeletonized pedicle on the subsurface of a skin flap was improved when microvascular distal arteriovenous shunt anastomoses were carried out, in comparison with distal ligation [56]. The authors concluded that the shunt anastomosis model, representing maximal blood flow, enabled tissue perfusion by the pedicle significantly earlier than the minimal blood flow model. In 2005, Hoang et al. [57] studied neo-vascularization in prefabricated flaps. The authors used Radiofluor X-ray and contrast agents injected in rabbits to show that a rich vascular tree can progressively create a bridge between implanted vessels on the recipient site and the flap’s own vessels. In this model, they showed that 20 days were needed to obtain mature neo-vascularization. Investigation of flap autonomization through progressive ischemia has also been investigated in rabbits, including work by Huang et al. which demonstrated that progressive restriction of flow through the pedicle via ligation resulted in higher rates of survival after pedicle division on POD 6 [58]. A number of studies have also investigated the effects of angiogenic agents on neo-vascularization, including the use of endothelial cell growth supplement, endothelial cell growth factor, tumor necrosis factor alpha, and adipose tissue-derived stromal cells [59,60,61,62]. These results largely mirrored those found in rats, demonstrating more rapid neo-vascularization when compared to controls, indicating that angiogenic agents may be effective in increasing the rate of autonomization and survival of fasciocutaneous flaps. 

**Part 2:**

Geoffrey G. Hallock is a pioneer reconstructive surgeon and one of the founding fathers of modern reconstructive surgery. He gave much consideration to fasciocutaneous flaps and developed an essential classification based on the type of vascularization [63]. Earlier, he tried to understand how to improve the reliability of dividing cross-finger flaps, which relied exclusively on capillary refill time after blocking the blood source [64]. He described the assessment of these flaps with a laser Doppler probe to measure flow changes at different time points and following pedicle compression. He showed that a preserved flow higher than 50% of the value before applying compression was correlated with full survival of the flap following division. In 2012, McGrath and her group [65] translated fluorescein angiography to patients to improve the reliability of groin flap division. They provided rare evidence of “early” flap autonomization allowing pedicle division after 3 to 4 weeks. Similarly, Galti et al. [66] used fluorescein to perform early division of a groin-to-hand flap at 14 days. A cutting-edge technique described by Furnas et al. in 1985, combining angiography, oximetry, and ischemic preconditioning of pedicled flaps (one groin flap and one cross-leg flap), allowed for the division of the bridge as early as 5 days after surgery. Similarly, George et al. [67] showed the early division of various pedicled flaps using progressive compression of the skin bridge in 1996. However, a majority of groin flap case series use a minimum of a 3-week delay prior to pedicle division [68,69,70], as initially described by McGregor [14]. Regarding lower limb reconstructions, the conventional autonomization delay is considered to be slightly longer. Even if some authors have described earlier time points [71], the cross-leg flap is mostly divided after at least 4 weeks [15,16,17], even in the most recent case series [18,72,73,74,75]. Modern technologies have brought interesting applications to the study of flap autonomization. Mucke et al. performed an interesting prospective clinical study in intra-oral free flaps using oxygen measurement technology and pedicle compression [76]. They showed that the recipient site location, flap type, and history of irradiation of the wound bed significantly influenced autonomization. They also confirmed flap autonomization in mucosal reconstructions, which was previously poorly explored. Another interesting approach was brought about by the advent of indocyanine green (ICG) angiography, which has been shown to be a better alternative to fluorescein [77,78]. Several teams used it to assess facial flap perfusion prior to pedicle division [79,80,81], but most of them still performed the second-stage surgery after 3 weeks, as indicated by the earliest descriptions of the forehead flap technique [5,7,8,82]. Still, a few authors tried to improve this ancestral technique’s efficiency by looking for early autonomization. Abdelwahab et al. [79] found no contra-indication for early pedicle division in nasolabial flap reconstruction (mean 23 days), and Surowitz [83] and Rudy [84] showed no complications in selected patients when decreasing neo-vascularization time to 14 and 7 days, respectively. All of these clinical reports address proof of autonomization, autonomization delay, and/or enhancement through ischemic preconditioning. The physiology of the process remains unknown, since no work clearly distinguishes between neo-angiogenesis and capillary re-permeabilization bridging the two vascular systems. Future studies could focus on these mechanisms, addressing critical gaps for future bioengineering applications. Finally, while the abovementioned studies prove the autonomization of fasciocutaneous flaps in clinical practice, it seems critical to highlight evidence of the early occurrence of this process. 

**Part 3:**

**Figure 2:**
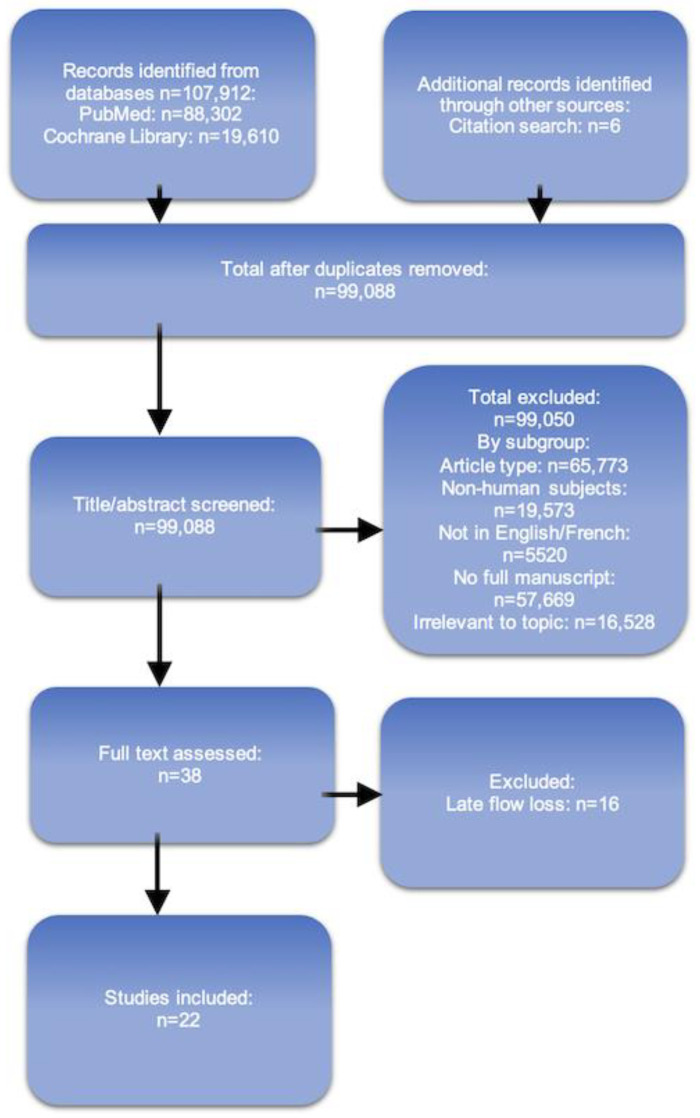
Reports of early fasciocutaneous flap autonomization. PRISMA Flowchart summarizing the number of included articles in the screening and selection process for Part 3. The last search was conducted on 15 June 2023.

Our systematic review identified 107,912 articles from the Pubmed and Cochrane databases (Figure 2). A total of 8830 duplicates were removed. Using the dedicated filters, the following numbers of articles fit the exclusion criteria: 65,773 articles were excluded because of the article type (other than case reports, case series, clinical studies and trials, letters, editorial, abstracts), 19,573 articles were excluded because of non-human subjects, 5520 articles were excluded because of the language, and 57,669 articles had no accessible full manuscript. Finally, 16,528 articles were irrelevant to the topic [describing flap revision, flap failure, flap survival later than (2 weeks for head, neck, and hands) and (3 weeks for other sites), muscle flaps, or not describing a precise day of flow discontinuity]. Six additional articles were found by manual cross-reference screening [85,86,87,88,89]. At the end of the screening process, we included 22 articles in the final analysis [84,85,86,87,88,89,90,91,92,93,94,95,96,97,98,99,100,101,102,103,104]; all were case reports or case series (Table 1). Table 2 displays flaps and patients’ characteristics. Fifty-two fasciocutaneous flaps with early disruption of the main blood source were analyzed from the included articles. The mean age of the included patients was 62.7 ± 16.5 years old. Most of the initial conditions leading to the pre-operative defect were tumors, among which carcinomas were the most frequent cause. Up to 23% of the patients had received radiotherapy before the fasciocutaneous flap surgery, and 21% had an ongoing local infection. Two patients were actively smoking. Overall, 82% of the flaps healed with no complication, while 15% had a partial loss. The mean delay before discontinuity (DBD) of the feeding source was 9.3 ± 4.5 days. 

When focusing on the anatomic recipient site, out of the 52 flaps, 13 were transposed in the oral cavity (mucosa), 29 were used for head and neck defect reconstruction, 6 were used for limbs, and 4 were used for the breast. We carried out a sub-group analysis based on these anatomic sites (Table 3). In the intra-oral/mucosa group, no partial loss was reported, with a mean DBD of 11 ± 4.3 days. In contrast, the breast group had 75% complications with partial loss, while the mean DBD was 7.8 ± 2.5 days. The head and neck and the limb group had intermediary outcomes, with 13.8% and 16.7% partial losses and a mean DBD of 7.76 ± 3.17 and 13.67 ± 7.23 days, respectively. Overall, no significant difference was found between groups for DBD. Breast flaps had significantly higher rates of partial loss when compared with intra-oral flaps (*p* = 0.009; Fisher’s exact *t*-test). 

Another post hoc analysis was performed to assess the impact of the vascularization type [direct vs. indirect vessels (musculocutaneous branches), Table 4]. In this series, axial flaps had significantly lower DBD than perforator and septal flaps (*p* = 0.002 and *p* = 0.0138, respectively) but showed no partial loss. It was our intention to compare the outcomes depending on the size of the flap’s skin paddle, but missing and non-homogenous data were too important to provide significant results. Similarly, the flap thickness was missing in most reports and was not included in the analysis. Up to 50% (n = 6) of the patients who had received radiotherapy before the flap surgery experienced partial flap loss. A sub-group analysis (Fisher’s exact *t*-test) found a significant increase in flap partial loss in comparison with patients with no pre-operative radiotherapy [*p* = 0.006; odds ratio 12.5; CI 95% (1.01; 66.58)].

It is worth noting that Wolff et al. showed partial autonomization of fasciocutaneous flaps used as free flaps while perfused with an extracorporeal perfusion system. This novel reconstruction technique was achieved by perfusing the flaps with diluted autologous blood for 4–6 days before interruption. Despite the novelty of the technique described by the authors, we decided to include their cases as examples of strong evidence of the autonomization process and its delay. 

## 4. Discussion

The principle of autonomization is not unanimously recognized among the authors. Some are reluctant to accept it and consider that a flap remains indefinitely dependent on its pedicle [105]. This belief is supported by described cases of flap necrosis several years after surgery, following a delayed division of the pedicle [92,106]. Other authors warn against risk factors such as irradiation, atherosclerosis, and smoking [76,92,105]. However, the advent of modern monitoring techniques, such as indocyanine green angiography and contrast-enhanced ultrasonography, helped confirm the acquired independence of the flaps from their initial blood source [4,107,108]. 

This literature review provides additional reassurance to the reconstructive surgeon regarding the vulnerability of fasciocutaneous flaps. The case reports included in Part 3 show that flaps used for face reconstruction can be detached from the pedicle as early as one week, as opposed to the commonly accepted 3-week delay [5,6,7]. Interestingly, intra-oral/mucosal flaps seemed to show important potential for early autonomization, with 100% full survival with a mean DBD of 11 days (SD 4.3 days]. This is confirmed by the study conducted by Mucke et al. [76] that proved the neo-vascularization of these flaps using advanced imaging. For limb reconstruction, where the commonly accepted times can range up to 6 weeks, our review suggests that a period of 15 days can be enough in a non-irradiated area. Our sub-group analysis, indeed, found a higher partial flap loss rate in irradiated patients, according to previous studies [109]. We deliberately chose to highlight the shortest duration of autonomization, selecting restrictively short durations of autonomization as an inclusion criterion. The objective was to provide the surgeon with evidence of the rapid nature of this process. Some authors described the full survival of Deep Inferior Epigastric Perforator (DIEP) flaps for breast reconstruction after pedicle resection several years later [110,111]. In our review, the three cases describing the early loss of blood flow (mean of 7.8 days) in free flaps for breast reconstruction showed systematic complications but partial survival. Further studies should focus on flap autonomization in breast reconstruction, since these flaps are not only used to transfer skin, but also to provide volume in the modern era of areola- and skin-sparing mastectomies [112]. The cases included in this review did not allow for addressing the impact of the flap’s volume and thickness, which could be a critical factor in the autonomization delay. A second limit was the lack of data on the skin paddles’ sizes. This can change flap outcomes due to a higher or lower surface area in contact with the wound bed and margins, thus influencing the possible area of neo-vascularization. Even if flaps used for face and intra-oral reconstructions are usually smaller than free flaps used for breast or limb reconstruction, any interpretation of these differences from this review would be risky due to the marked difference in vascularization of the aforementioned sites. Moreover, the post hoc sub-group analyses could have led to an increased statistical bias, and the resulting conclusions should be considered carefully. 

This paper comes as an update to the article published by Yoon et al. in 2016 [113]. We specifically focused on muscle-sparing flap surgery, which is becoming the gold standard in reconstructive plastic surgery. We also aimed to focus on early autonomization that could lead to a change in current practice. Moreover, we included cases described by Wolff et al. [19,102,104], which were novel since they consisted of intermittently perfused flaps with an extracorporeal system. This is the first clinical in vivo description of flap autonomization enhancement through intermittent ischemic preconditioning. However, their series showed that a majority of flaps developed ischemic complications, with partial necrosis and/or epidermolysis, thus indicating that their innovative techniques need further optimization. In short, this work provides information on the postoperative delay in the autonomy of fasciocutaneous flaps, as well as certain factors that may influence this duration and therefore should be taken into account. As the level of evidence provided remains low, it is essential that other, more robust, studies be carried out with the objective of analyzing the time period required for flap neo-vascularization. 

These results are in addition to numerous research studies in preclinical models, including some of the earliest investigations into autonomization at the bench. An article by Payement et al. [114] examined the survival of flaps in a rat model after expansion of the pedicle, demonstrating that 50% of iliac island skin flaps remained viable 2 months after pedicle expansion. Further work in the field has continued to develop knowledge of autonomization, such as Mucke et al.’s [48,78] investigations of the minimal time to flap autonomization in rats, allowing for clinically negligible necrosis. They showed that, in this model, fasciocutaneous flaps could fully survive after pedicle ligation at 7 days postoperatively. They used laser spectrometry to bring an objective assessment of flap viability. They also showed that oxygen saturation, hemoglobin levels, blood flow, and blood velocity in the flap impacted its survival. 

Other studies investigated the role of neo-vascularization in flap survival, with a particular focus on factors that promote the process. Semashko [45] and later, our team [44], found higher flap survivability in the most distant part from the feeding vessels (Figure 1). This proximal–distal gradient could be due to the ischemic condition of the tip, which is the least-perfused portion of a flap. Therefore, ischemia could be the first critical factor influencing this phenomenon. Vourtsis et al. examined the impact of subdermal injections of vascular endothelial growth factor (VEGF) in a random skin flap rat model, observing approximately double the flap survival rate in the VEGF group as compared to the control [115]. Histological examination of the flaps demonstrated angiogenesis in the experimental group, suggesting that VEGF treatment hastened the process of autonomization. Pretreatment of the flap to promote autonomization has also been investigated, such as Efeoğlu et al.’s paper investigating the subcutaneous application of omentin to flaps before their elevation [116]. Omentin was injected at one week preoperatively in one group of rats and both 2 days before and on the day of surgery in another group. The authors found that omentin increased the endothelial nitric oxide synthase expression, the viable area of the flap after surgery, the thickness of the epidermal layer of the flap, and the level of angiogenesis postoperatively. These works demonstrate the considerable advances being made at the bench and the importance of further investigation and eventual translation of this knowledge to the clinic. 

The contribution of additional data could lead to changes in the management of patients by allowing an update of techniques and reducing the time required to perform transient pedicle reconstruction. In addition, many authors describe techniques to make conventional microsurgical delayed reconstructions more reliable. These multi-step procedures could benefit from simplification resulting from a reduction in the necessary delay. Another benefit that may result from these data is the improvement in the reliability of extracorporeal machine-perfused flap reconstruction techniques [117,118], as initially described by Wolff [102]. Their pioneering work requires optimization to decrease the observed complication rate, yet the total perfusion time (4 to 6 days) deserves attention.

**Figure 3:**
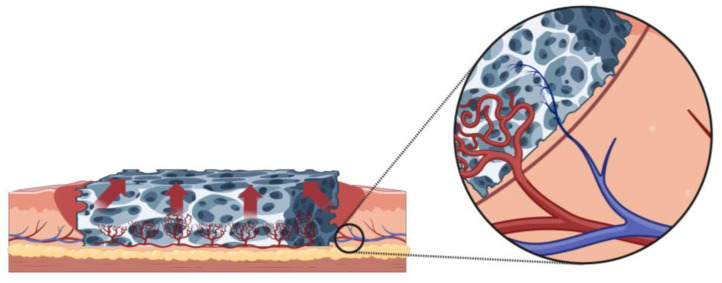
Application of the autonomization process in tissue engineering for decellularized scaffold revascularization. The hypothesis is that the scaffold can be used to host angiogenesis from a recipient wound bed, making it possible to revascularize the entire depth, ensuring long-term survival of cells seeded during recellularization protocols.

Finally, in the realm of tissue engineering and complex scaffold recellularization, the revascularization process becomes of major importance. This has been emphasized as one of the main limits in bioengineered livers [119,120,121]. Stabler et al. [122] pointed out the limited viability of transplanted engineered lungs in a rodent model, due to the lack of reconstruction of the endothelium lining. Their literature review highlights the importance of vascular function and not only vascular cell reseeding. In composite scaffolds, Zhang et al. [123] recently showed better human umbilical vein endothelial cells (HUVECs) growth in penile scaffolds conjugated with heparin in a mouse model. However, their findings are preliminary and the lack of complexity of the used model makes further studies necessary. Alternatively, Nyirjesy et al. [124] performed in vivo implantation of decellularized and composite tracheal scaffolds and showed successful neovessel formation, with tubular vessels lined with endothelial cells at 1 month. This acts as an interesting proof-of-concept of using the autonomization process discussed in this manuscript. This promising approach could lead to engineered seeded scaffolds receiving in vivo revascularization to ensure long-term survivability of the resulting recellularized matrix by providing reliable and full-thickness blood flow (Figure 3). Our group is actively working on exploring this hypothesis for complex decellularized scaffolds in reconstructive surgery applications, and further work should be performed in order to reach the critical milestone of bioengineered composite flap reconstruction.

## 5. Conclusions

The autonomization process is widely used in reconstructive surgery. Autonomization times for complex, composite structures such as fasciocutaneous flaps appear to be shorter than is commonly accepted in practice. These data are already being used in the development of new cutting-edge reconstructive techniques, such as flap reconstruction using extracorporeal perfusion. Application in tissue engineering therefore seems to be the next step, ultimately enabling universal reconstructions based on recellularized scaffolds. 

## Figures and Tables

**Table 1:** Articles included in the systematic review analysis (Part 3).

PubMed ID (Reference #)	Year	Author	Article Type
6190527 [90]	1983	Rothaus	Case report
7493792 [86]	1995	Skbric	Case report
10513925 [89]	1999	Amato	Case report
11562041 [87]	2001	Ceulemans	Case report
123797 [91]	2002	Godden	Case report
12404130 [92]	2002	Salgado	Case series
12946680 [93]	2003	Castling	Case report
15013552 [94]	2004	Kissun	Case report
15074725 [95]	2004	Ribuffo	Case report
15908077 [96]	2005	Burns	Case series
18495566 [88]	2008	Branford	Case report
19446514 [97]	2009	Enajat	Case report
20878730 [98]	2010	Chubb	Case report
20175197 [99]	2010	Wise	Case report
22186589 [100]	2012	Nelson	Case series
* [85]	2013	Hindocha	Case report
25643188 [101]	2015	Granzow	Case series
26752222 [102]	2016	Wolff	Case series
28642191 [103]	2017	Wolff	Case report
31874806 [104]	2020	Wolff	Case series
32565139 [84]	2021	Rudy	Case series

* No PMID, DOI: https://doi.org/10.1308/204268513X13776914744871.

**Table 2:** Included patients and characteristics of the flap procedure, loss of principal blood source, and outcomes.

1st Author	Study Type	Age	Initial Pathology	Recipient Site	RxTh *	Smoking Status	Diabetes	Local Infection	Flap	Flap Classification	Flap Size	DBD *	Type of BD *	Partial Flap Loss
Amato	Case report	68	SCC	Mandible	Yes	Previous	NA	No	Scapular	Perforator/Free		4	Venous	No
Branford	Case report	48	Trauma	Heel	No	No	Yes	No	RFF	Septal/Free	6 × 5	26	Pedicle	Minor necrosis
Burns	Case series	59	SCC	Tongue	No	NA	NA	Yes	RFF	Septal/Free		19	Veinous	No
Burns	Case series	69	SCC	Tongue	No	NA	NA	No	RFF	Septal/Free		11	Veinous	No
Burns	Case series	49	Carcinoma	Tongue	NA	NA	NA	No	RFF	Septal/Free		6	Pedicle	No
Castling	Case report	52	Adenoic cystic carninoma	Tongue	No	NA	NA	Yes	RFF	Septal/Free		9	Pedicle	No
Ceulemans	Case report	65	Trauma	Ankle	No	No	No	Yes	TDAP	Perforator/Free		18	Pedicle	No
Chubb	Case report	50	DCIS	Breast	Yes	No	NA	No	SGAP	Perforator/Free	400 g	7	Pedicle	Epidermolysis, 10% Necrosis
Enajat	Case report	64	Carcinoma	Breast	Yes	No	NA	No	SIEA	Perforator/Free		11	Pedicle	25% Necrosis
Felcht	Case series	75	Carcinoma	Nasal tip	No	NA	NA	No	Forehead	Axial/Pedicled	6.3 cm^2^	7	Pedicle	No
Felcht	Case series	70	Carcinoma	Nasal dorsum	No	NA	NA	No	Forehead	Axial/Pedicled	7.5 cm^2^	7	Pedicle	No
Felcht	Case series	84	Carcinoma	Nasal tip	No	NA	NA	No	Forehead	Axial/Pedicled	6.3 cm^2^	7	Pedicle	No
Felcht	Case series	80	Carcinoma	Nasal tip	No	NA	NA	No	Forehead	Axial/Pedicled	5.8 cm^2^	7	Pedicle	No
Felcht	Case series	79	Carcinoma	Nasal tip	No	NA	NA	No	Forehead	Axial/Pedicled	9 cm^2^	7	Pedicle	No
Felcht	Case series	78	Carcinoma	Nasal dorsum	No	NA	NA	No	Forehead	Axial/Pedicled		7	Pedicle	No
Felcht	Case series	87	Carcinoma	Nasal tip	No	NA	NA	No	Forehead	Axial/Pedicled	6.5 cm^2^	7	Pedicle	No
Felcht	Case series	60	Carcinoma	Nasal sidewalls	No	NA	NA	No	Forehead	Axial/Pedicled	6.9 cm^2^	7	Pedicle	No
Felcht	Case series	90	Carcinoma	Nasal tip	No	NA	NA	No	Forehead	Axial/Pedicled	5.5 cm^2^	7	Pedicle	No
Felcht	Case series	87	Carcinoma	Nasal tip	No	NA	NA	No	Forehead	Axial/Pedicled	3.4 cm^2^	8	Pedicle	No
Felcht	Case series	76	Carcinoma	Nasal tip	No	NA	NA	No	Forehead	Axial/Pedicled	6.3 cm^2^	11	Pedicle	No
Felcht	Case series	87	Carcinoma	Nasal ala	No	NA	NA	No	Forehead	Axial/Pedicled	7.3 cm^2^	7	Pedicle	No
Godden	Case report	40	SCC	Tongue	No	NA	NA	Yes	RFF	Septal/Free	NA	9	Pedicle	No
Granzow	Case series	76	SCC	External Cheek	No	No	NA	No	Fibular	Septal/Free	20 × 16	17	Arterial	No
Granzow	Case series	39	Ameloblastoma	Intra-oral Cheek	No	No	NA	No	Fibular	Septal/Free	27 × 10	11	Pedicle	No
Hindocha	Case report	55	SCC	Buccas mucosa	No	Yes	No	No	RFF	Septal/Free		12	Arterial	No
Kissun	Case report	35	SCC	Tongue	Yes	NA	NA	Yes	Radial Forearm flap	Septal/Free		6	Pedicle	No
Nelson	Case series	49	Cancer	Breast	NA	NA	NA	No	DIEP	Perforator/Free		5	Venous	Yes
Nelson	Case series	52	Cancer	Breast	NA	NA	NA	No	SGAP	Perforator/Free		8	Venous	No
Ribuffo	Case report	42	Trauma	Ankle	No	NA	NA	No	RFF	Septal/Free	8 × 4	11	Venous	No
Rothaus	Case report	17	Trauma	Heel	No	No	No	No	Groin Flap	Axial/Free	9 × 9	9	Arterial	No
Rudy	Case series	87	Melanoma in situ	L Nasal dorsum	No	No	No	No	Forehead	Axial/Pedicled		7	Pedicle	No
Rudy	Case series	77	BCC	L Nasal Ala	No	No	No	No	Forehead	Axial/Pedicled		7	Pedicle	No
Rudy	Case series	55	BCC	L Nasal tip	No	No	No	No	Forehead	Axial/Pedicled		7	Pedicle	No
Rudy	Case series	74	BCC	L Nasal tip	No	No	No	No	Forehead	Axial/Pedicled		7	Pedicle	No
Rudy	Case series	52	BCC	R Nasal lateral wall	No	No	No	No	Forehead	Axial/Pedicled		7	Pedicle	No
Rudy	Case series	51	BCC	R Nasal tip	No	No	No	No	Forehead	Axial/Pedicled		7	Pedicle	No
Rudy	Case series	58	BCC	R Nasal Ala	No	No	No	No	Forehead	Axial/Pedicled		7	Pedicle	No
Rudy	Case series	65	BCC	R Nasal Ala	No	No	No	No	Forehead	Axial/Pedicled		7	Pedicle	No
Rudy	Case series	89	BCC	R nasal tip	No	No	No	No	Forehead	Axial/Pedicled		7	Pedicle	No
Salgado	Case series	62	SCC	Tongue	No	No	No	Yes	Fibular	Septal/Free		8	Pedicle	No
Salgado	Case series	38	Trauma	Tongue	No	No	No	Yes	Fibular	Septal/Free		10	Pedicle	No
Salgado	Case series	61	SCC	Mouth	No	No	Yes	Yes	Fibular	Septal/Free		13	Pedicle	No
Salgado	Case series	47	SCC	Mouth	Yes	No	No	Yes	Fibular	Septal/Free		20	Pedicle	No
Skbric	Case report	37	Trauma	Heel	No	NA	NA	No	RFF	Septal/Free		12	Arterial	No
Wise	Case report	69	SCC	Tongue	Yes	Previous	No	Yes	ALT	Perforator/Free		9	Pedicle	No
Wolff	Case report	57	Secondary defect	Shoulder	Yes	NA	NA	No	ALT	Perforator/Free	13 × 8	6	Pedicle	No
Wolff	Case series	52	SCC	Chin	Yes	NA	NA	No	ALT	Perforator/Free	25 × 8	18	Pedicle	No
Wolff	Case series	77	Carcinoma	Neck	Yes	NA	NA	No	ALT	Perforator/Free	14 × 9	6	Pedicle	Yes: epithelial + hilum
Wolff	Case series	60	Carcinoma	Cheek	No	NA	NA	No	ALT	Perforator/Free	7 × 6	6	Pedicle	Yes: epithelial
Wolff	Case series	76	CUP syndrom	Neck	Yes	NA	NA	No	RFF	Septal/Free	8 × 6	5	Pedicle	Yes: epidermis + dermis
Wolff	Case series	70	Glioblastoma	Occipital scalp	Yes	NA	NA	Yes	RFF	Septal/Free	14 × 9	4	Pedicle	Yes: 80%
Wolff	Case series	66	SCC	Cheek	Yes	No	NA	No	Fibular (septal)	Septal/Free	6 × 4	13	Pedicle	No

* BD: Blood discontinuity; DBD: Delay of blood discontinuity; RxTh: Radiotherapy history; SCC: Squamous Cell Carcinoma; DCIS: Ductal Carcinoma In Situ; BCC: Basal Cell Carcinoma; CUP: Cancer of Unknown Primary; L: Left; R: Right; RFF: Radial Forearm Flap; TDAP: Thoracodorsal Artery Perforator flap; SGAP: Superior Gluteal Artery Perforator flap; SIEA: Superficial Inferior Epigastric Artery flap; DIEP: Deep Inferior Epigastric Artery Perforator flap; ALT: Anterolateral Thigh flap.

**Table 3:** Sub-group analysis by anatomic location.

Flap Location	Number of Flaps	Day of Discontinuity (Mean ± SD)	Partial Loss n (Mean)	Earliest Full Autonomization (Days)
Head/Neck	29	7.76 ± 3.17	4 (14%)	4
Intra-oral	13	8.00 ± 4.06	0 (00%)	6
Limb	6	13.67 ± 7.23	1 (17%)	6
Breast	4	7.75 ± 2.50	3 (75%)	8
Total	52	9.25 ± 4.46	8 (15%)	4

**Table 4:** Sub-group analysis by flap pedicle type.

Vascularization Type	Number of Flaps	Day of Discontinuity	Partial Loss n (Mean)
		(Mean ± SD)	
Indirect (Musculocutaneous)	12	8.67 ± 4.74	4 (33%)
Direct Septal	18	12.00 ± 5.56	3 (17%)
Direct Axial	22	7.31 ± 0.89	0 (00%)
Total	52	9.25 ± 4.46	8 (15%)

## Data Availability

No data were created for this review article. All data analyzed were included in the manuscript. All data or information can be provided on demand by the corresponding author.

## Appendix A

**Table A1:** ROBIS tool: Assessment of Bias Risk.

	Concern	Rationale for Concern
1. Concerns regarding specification of study eligibility criteria	Study size was not restriced.	Studies with small sample sizes were included due to limited data availability.
2. Concerns regarding methods used to identify and/or select studies	Eligibility criteria may have excluded pertinent papers in other languages.	Papers not in English or French were excluded.
3. Concerns regarding methods used to collect data and appraise studies	Not all study characteristics were available for review.	Some papers did not include potentially relevant information.
4. Concerns regarding the synthesis and findings	Synthesis may not have included all the studies it should have.	Some relevant studies may not have been found during the search process.
**RISK OF BIAS IN THE REVIEW**		
Describe whether conclusions were supported by the evidence: the conclusions were supported by the evidence gathered.		
A. Did the interpretation of findings address all of the concerns identified in Domains 1 to 4? (Y)/PY/PN/N/NI B. Was the relevance of identified studies to the review’s research question appropriately considered? (Y)/PY/PN/N/NI C. Did the reviewers avoid emphasizing results on the basis of their statistical significance? (Y)/PY/PN/N/NI		
Risk of bias in the review RISK: (LOW)/HIGH/UNCLEAR Rationale for risk: Bias is unavoidable, but foreseeable risks of bias were mitigated.		
